# Penoscrotal Low-grade fibromyxoid sarcoma, A case report

**DOI:** 10.1016/j.eucr.2023.102499

**Published:** 2023-07-05

**Authors:** Abbas Basiri, Parham Montazeri, Mehdi Dadpour

**Affiliations:** Shahid Beheshti university of medical sciences, Shahid Labbafinejad medical center, Urology and Nephrology research center, Tehran, Iran

**Keywords:** Low-grade fibromyxoid sarcoma, Sarcoma, Penile sarcoma

## Abstract

Low-grade fibromyxoid sarcoma (LGFMS) is a subtype of sarcoma that commonly arises from the deep soft tissue. We present a case of LGFMS originated from the penoscrotal junction area, which highlights the unusual site of LGFMS presentation.The patient presented with a mass in the left base of the penis, which was resected and the pathology report was compatible with LGFMS. However, local recurrence near the primary tumor site was detected 3 months postoperatively, and re-excision confirmed the same diagnosis. This is the first report of LGFMS in the penoscrotal junction area as an uncommon site of this tumor.

## 1 Introduction

Low-grade fibromyxoid sarcoma (LGFMS) is a rare subtype of sarcoma that was described first by Evans in 1987.^1^ The most common tumor sites are the deep soft tissue of the limbs or trunk.^2^ The cause is unknown and the incidence is 0.18 per million, representing 0.6% of all sarcomas.^3^ According to the T1/T2 signal, MRI techniques are more useful than CT to detect the fibrous and myxoid components of the tumor.^4^ The gold standard treatment for localized diseases is marginally free surgical resection. Post op radiotherapy may improve local recurrence free surveillance.^5^ While Local recurrence and unusual distribution of metastatic disease are probable but there is no consensus about managing these situations. Re-excision may be considered in selected cases. Radiotherapy with or without adjuvant chemotherapy may be used when surgery is not feasible.^5^

There are a few reports of LGFMS in the genitalia area including left inguinal and scrotum in the literature which were managed with surgical resection, but to the best of our knowledge this pathology has not been reported in the penile tissue previously.^6^^,^^7^ Here we report a case of LGFMS in the base of penis at the penoscrotal junction area. this lesion and its recurrence were both managed surgically.

## 2 Case presentation

**Fig. 1:**
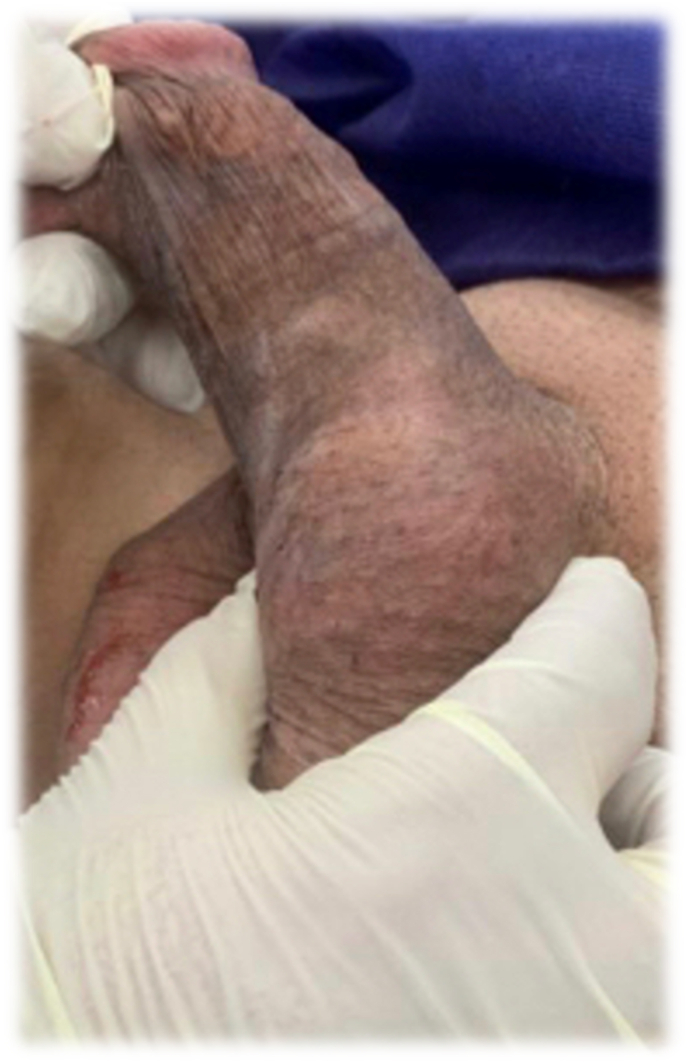
A firm mass in the base of penis above the left testis without pain and other symptoms.

**Patient information and medical history**: A 59-year-old man presented with a firm painless 3 cm mass in the left base of his penis at the penoscrotal junction area that has increased in size since 2 years ago (Fig. 1). There was no complaint of urinary symptoms and all other past medical history and physical examination were unmarkable.

**Fig. 2:**
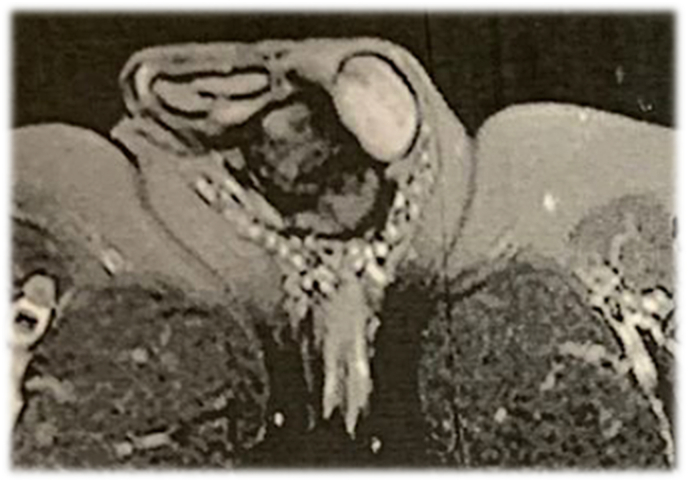
Pre-operation MRI that shows a low T1 high T2 subcutaneous lesion measuring 27*21 mm in left side of penis.

**Diagnosis and intervention:** Ultrasound study revealed a 17*30 mm hypoechoic solid lesion with clear borders and a brief internal flow on the left base of the penile shaft. Magnetic resonance imaging (MRI) without contrast showed a low T1 high T2 subcutaneous lesion measuring 27*21 mm in left side of penis which was compatible to a subcutaneous cyst (Fig. 2). There was no evidence of malignancy in cystourethroscopy evaluation. The pathology of core needle biopsy was consistent with LGFMS.

Considering all above, the patient was scheduled for surgical intervention. under general anesthesia and lithotomy position the lesion was completely resected with a 2mm macroscopic margin.

**Fig. 3:**
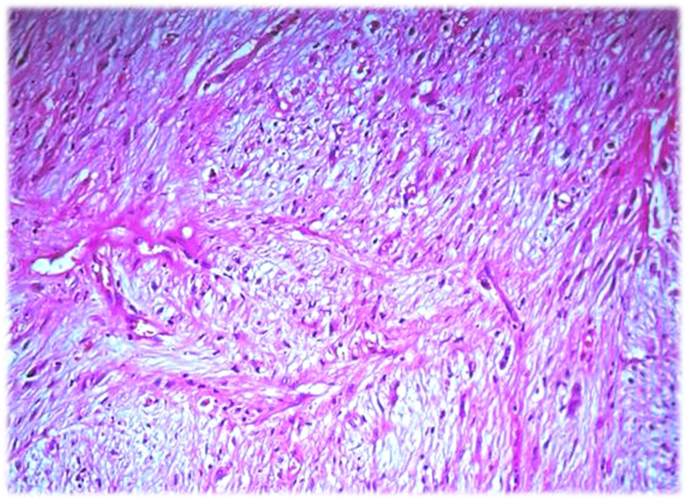
Histological image of the low grade fibromyxoid sarcoma showing an admixture of collagenized and hypocellular myxoid zones with a network of small size vessels.

On microscopic examination, Sections showed a neoplasm composed of intersecting fascicles of rather monotonous spindle cells with indistinct border, eosinophilic cytoplasm, oval shape nuclei and small nucleoli. The tumor revealed low to moderate cellularity. Mitotic figures were rare and no evidence of necrosis was seen. Few scattered moderately atypical cells were seen. The stroma was myxoid to collagenous which contained a network of small size vessels (Fig. 3). Immunohistochemistry staining revealed positive immunoreactivity for Vimentin and negative reaction for smooth muscle actin (SMA), h-caldesmon, desmin, S-100, CD34, CD99, HMB-45 CK5/6 and calretinin. Ki67 was positive in about 1% of tumoral cells. The diagnosis was LGFMS and all of the surgical margins were microscopically free of tumor.

**Fig. 4:**
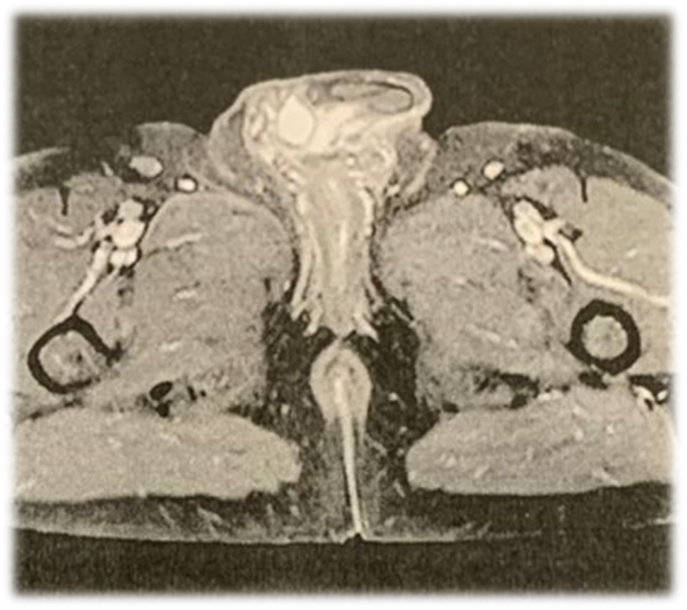
MRI taken 3 months after surgery that shows a low T1 high T2 sharp border enhancing 21*16 mm right penile root mass near the corpus spongiosum. Recurrence of known patient pathology (LGFMS) was suggested.

**Follow up:** About 2cm mass was detected near the site of surgery after 3 months follow-up. MRI study showed a low T1 high 12 sharp border enhaneing 21*16 mm right penile root mass near the corpus spongiosum (without obvious invasion) that remanent or recurrence of known patient pathology (LGFMS) was suggested (Fig. 4).

With diagnosis of local recurrence, re-excision was performed and pathology evaluation confirmed the previous diagnosis. One year follow-op revealed no evidence of local recurrence or distant metastasis.

## 3 Discussion

LGFMS is a rare subtype of sarcoma that commonly arise from the deep soft tissue. Although the deep soft tissue of the limbs or trunk is considered as the most prevalent area of the tumor, there are some unusual sites reported in the literature.^2^

LGFMS was reported in left inguinal area and in the right side of scrotum by Chitayat S et al., in 2020 and Sugino F et al., in 2022, respectively.^6^^,^^7^

To the best of our knowledge, our case is the first report of LGFMS in the penile tissue at the penoscrotal junction area.

Despite its low-grade appearance and slow-growing and asymptomatic behavior, local recurrence and distant metastasis may be probable. The tumor is generally seen as a single, well-circumscribed lesion, although after recurrence it can present as multiple infiltrative masses.^8^ In this presented case we observed local recurrence 3 months post operatively which did not occur in the same side of the primary tumor but very close in the opposite side of the penile shaft. Chitayat S et al. also reported local recurrence after surgical removement of left inguinal LGFMS.^6^ Folpe et al. reported 9% of local recurrence of LGFMS after surgery in a large case series.^9^^,^^10^ Guillou et al. reported 21% recurrence rate in their series.^11^

Definitive diagnosis of LGFMS depends on histopathological examination. Immunohistochemistry can help narrow the differential diagnoses and the diagnostic marker of LGFMS is MUC4. The main differential diagnoses are fibromatosis, fibrosarcoma, myxoid neurofibroma, myxofibrosarcoma, nodular fasciitis, malignant peripheral nerve sheath tumor, myxoid dermatofibrosarcoma.^9^^,^^10^

The standard treatment for LGFMS is surgical resection with negative margins for localized disease. However, local recurrence was observed in spite of negative surgical margin of the localized disease in our case. And it's unclear whether adjuvant therapy might benefit patients with recently diagnosed LGFMS.^3^

## 4 Conclusion

In conclusion, this is the first report of LGFMS in the penoscrotal junction area as an uncommon site of this tumor which was incidentally discovered in a 59-year-old man. Local recurrence occurred near to the previous tumor site 3 months post operatively in spite of negative surgical margin.

Long term follow up seems to be mandatory due to late metastases or local recurrence probability.

## Ethics approval and consent to participate

Informed consent from the patients was obtained in this report.

## Consent for publication

The consent was obtained from the patient to publish this report.

## Funding

No fund was used in this project.

## Authors' contributions

AB: project administration, conception, critical review; PM: drafting, reviewing, editing, data curation; MD: drafting, critical review.

## List of abbreviations

LGFMS: low grade fibromyxoid sarcoma
MRI: Magnetic resonance imaging
SMA: smooth muscle actin

## References

[1] Evans H.L. (1987). Low-grade fibromyxoid sarcoma: a report of two metastasizing neoplasms having a deceptively benign appearance. Am J Clin Pathol.

[2] Mohamed M., Fisher C., Thway K. (2017). Low-grade fibromyxoid sarcoma: clinical, morphologic and genetic features. Ann Diagn Pathol.

[3] Maretty-Nielsen K., Baerentzen S., Keller J., Dyrop H.B., Safwat A. (2013). Low-grade fibromyxoid sarcoma: incidence, treatment strategy of metastases, and clinical significance of the FUS gene. Sarcoma.

[4] Yue Y., Liu Y., Song L., Chen X., Wang Y., Wang Z. (2018). MRI findings of low-grade fibromyxoid sarcoma: a case report and literature review. BMC Muscoskel Disord.

[5] Chamberlain F., Engelmann B., Al-Muderis O. (2020). Low-grade fibromyxoid sarcoma: treatment outcomes and efficacy of chemotherapy. In Vivo.

[6] Chitayat S., Barros R., Ribeiro J.G. (2020). Case Report: an extremely rare occurrence of recurrent inguinal low-grade fibromyxoid sarcoma involving the scrotum. F1000Research.

[7] Sugino F., Ishida T., Tamaki M., Komeda H., Watanabe N., Tanaka T. (2022). Paratesticular low-grade fibromyxoid sarcoma: a case report. Hinyokika kiyo Acta Urologica Japonica.

[8] Hwang S., Kelliher E., Hameed M. (2012). Imaging features of low-grade fibromyxoid sarcoma (Evans tumor). Skeletal Radiol.

[9] Folpe A.L. (2002). Low-grade fibromyxoid sarcoma: a review and update. AJSP: Reviews & Reports.

[10] Folpe A.L., Lane K.L., Paull G., Weiss S.W. (2000). Low-grade fibromyxoid sarcoma and hyalinizing spindle cell tumor with giant rosettes: a clinicopathologic study of 73 cases supporting their identity and assessing the impact of high-grade areas. Am J Surg Pathol.

[11] Guillou L., Benhattar J., Gengler C. (2007). Translocation-positive low-grade fibromyxoid sarcoma: clinicopathologic and molecular analysis of a series expanding the morphologic spectrum and suggesting potential relationship to sclerosing epithelioid fibrosarcoma: a study from the French Sarcoma Group. Am J Surg Pathol.

